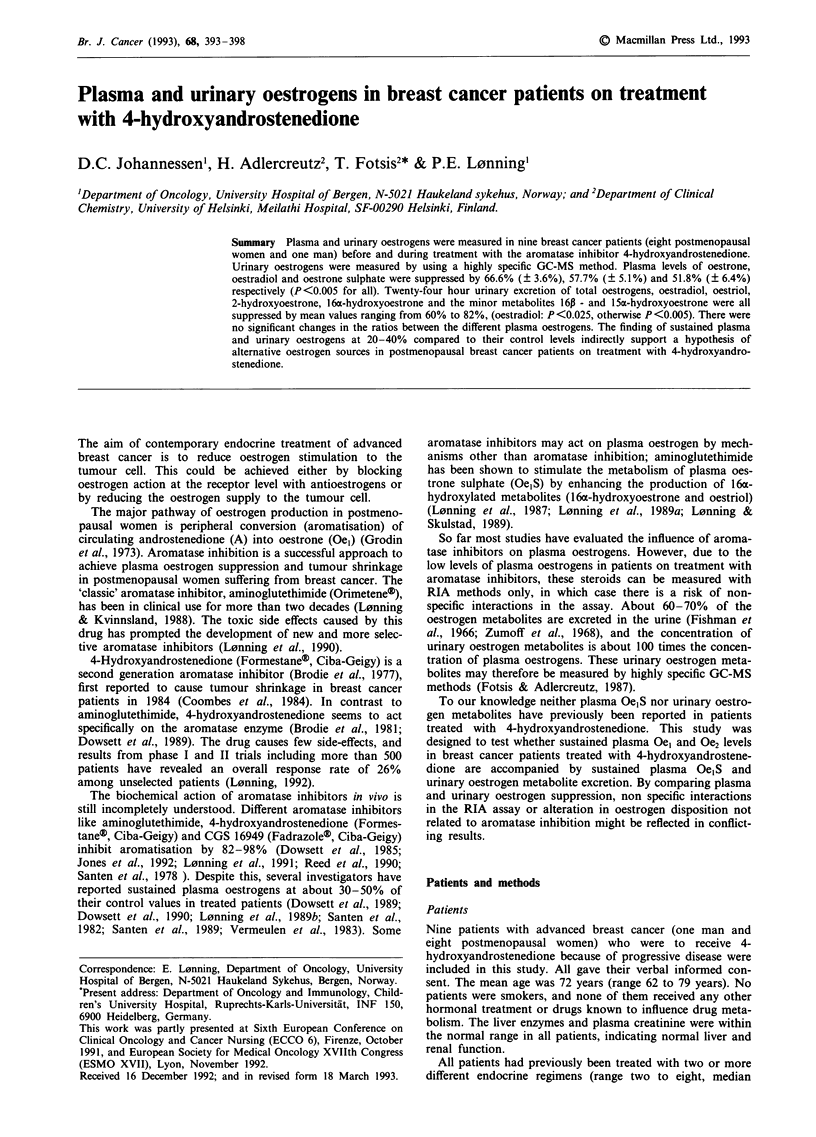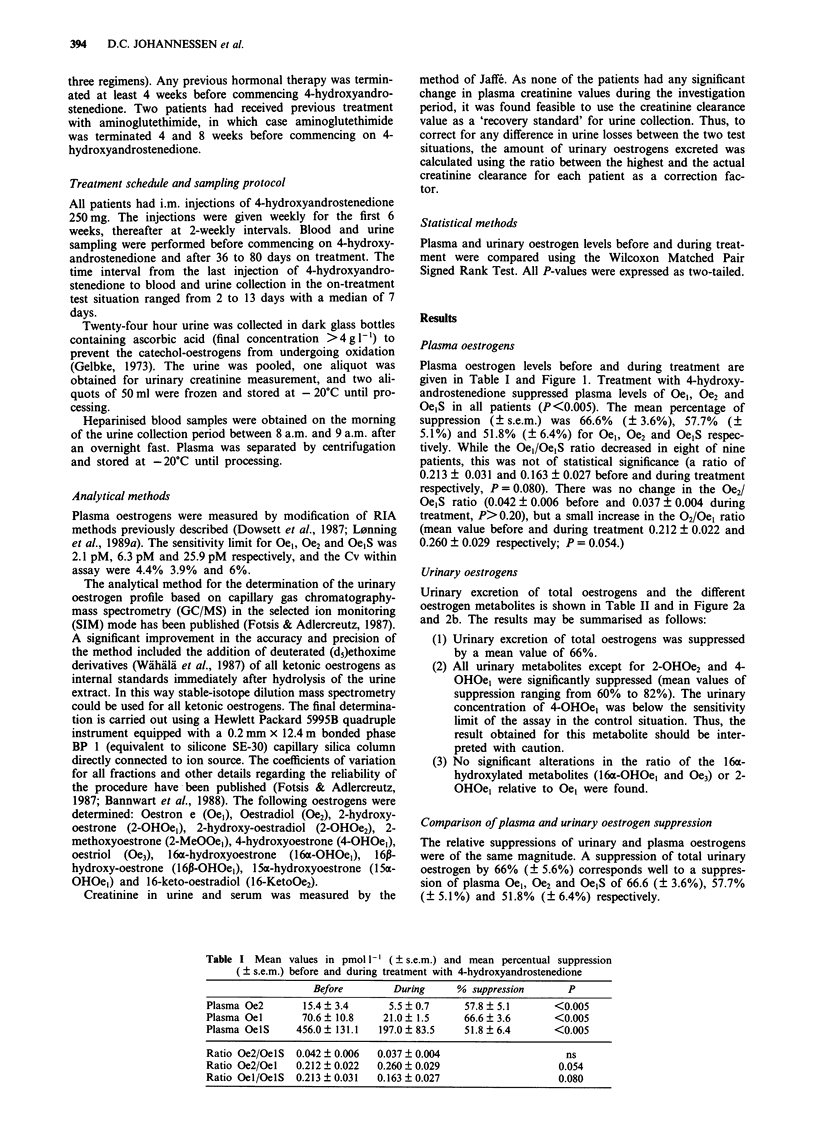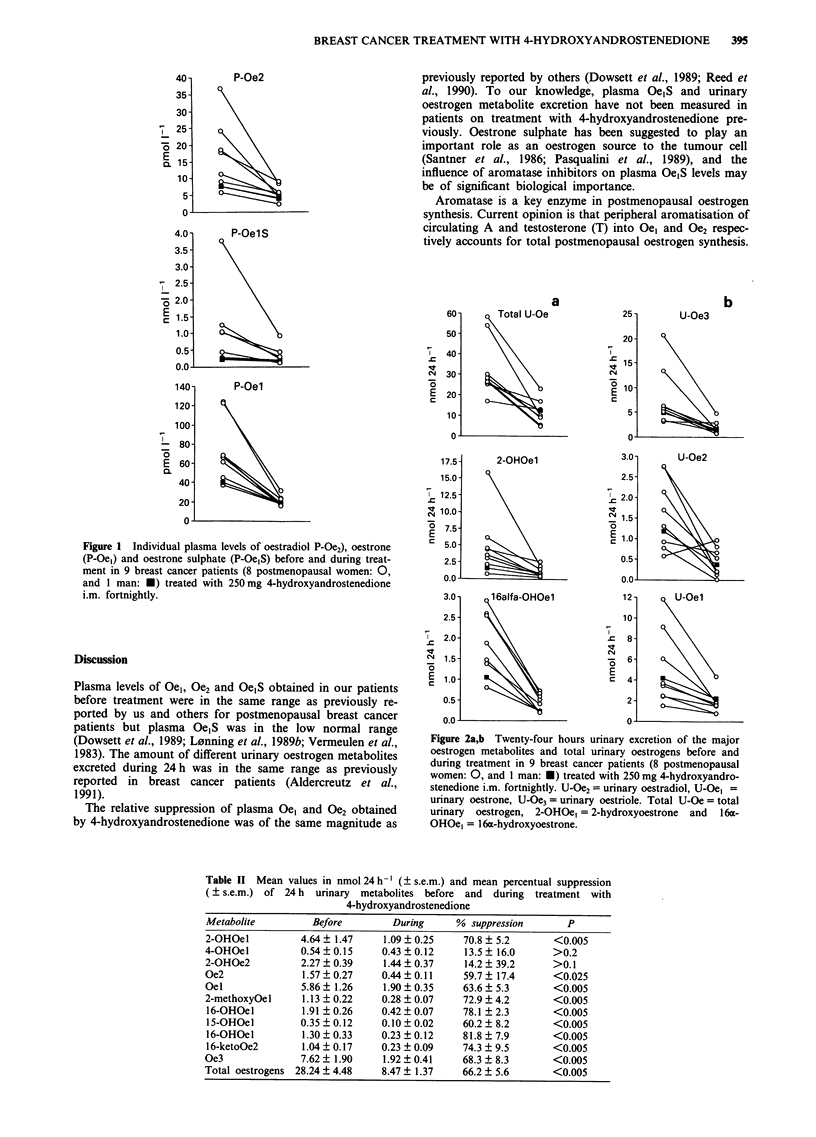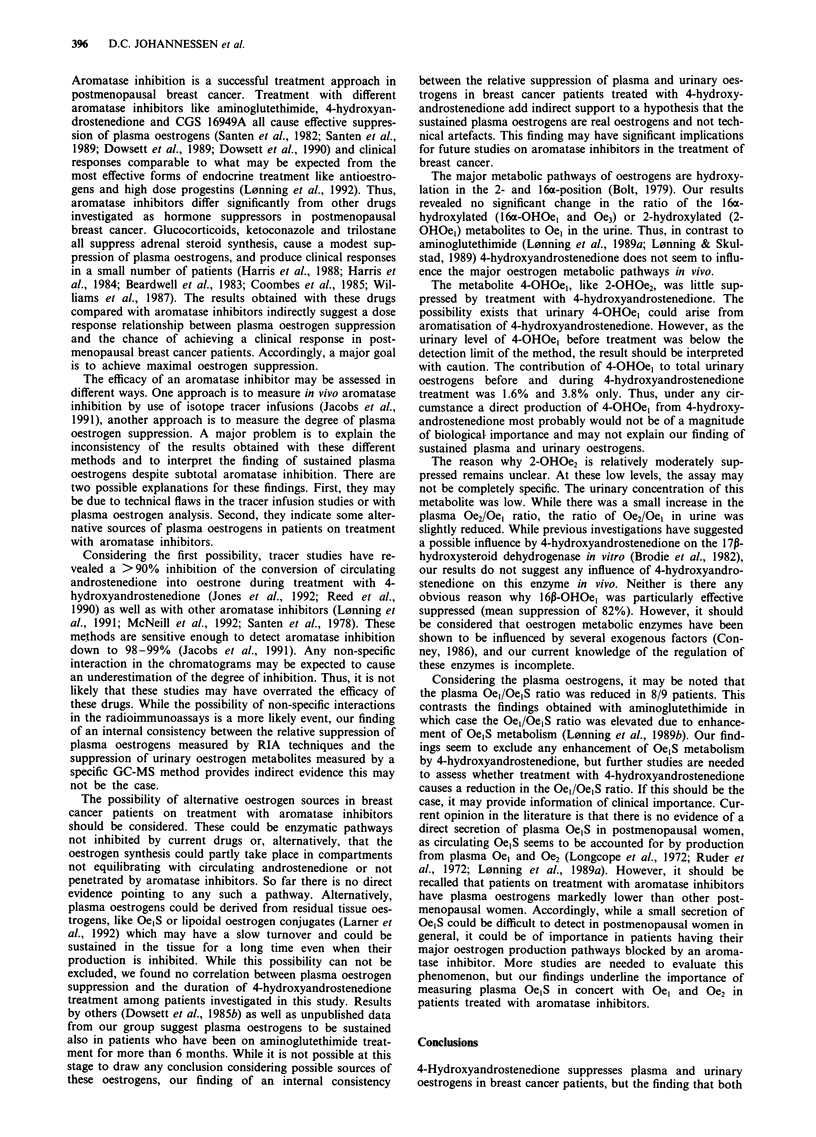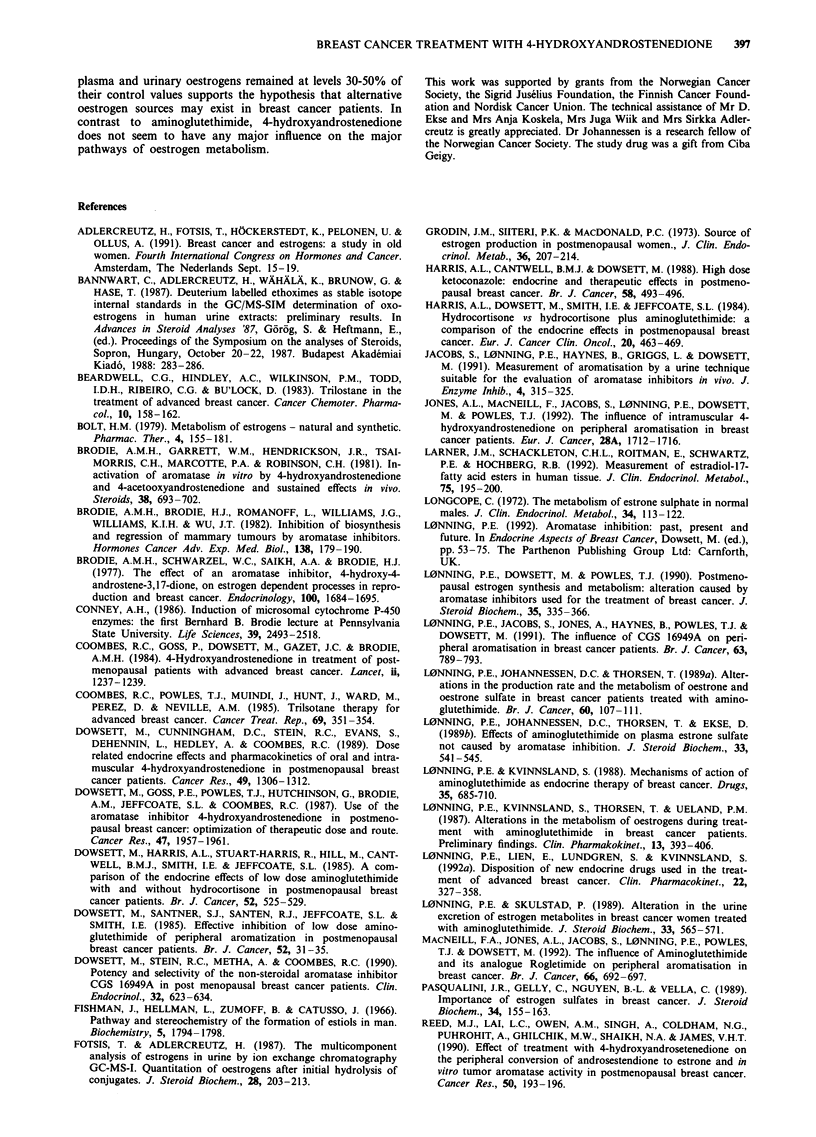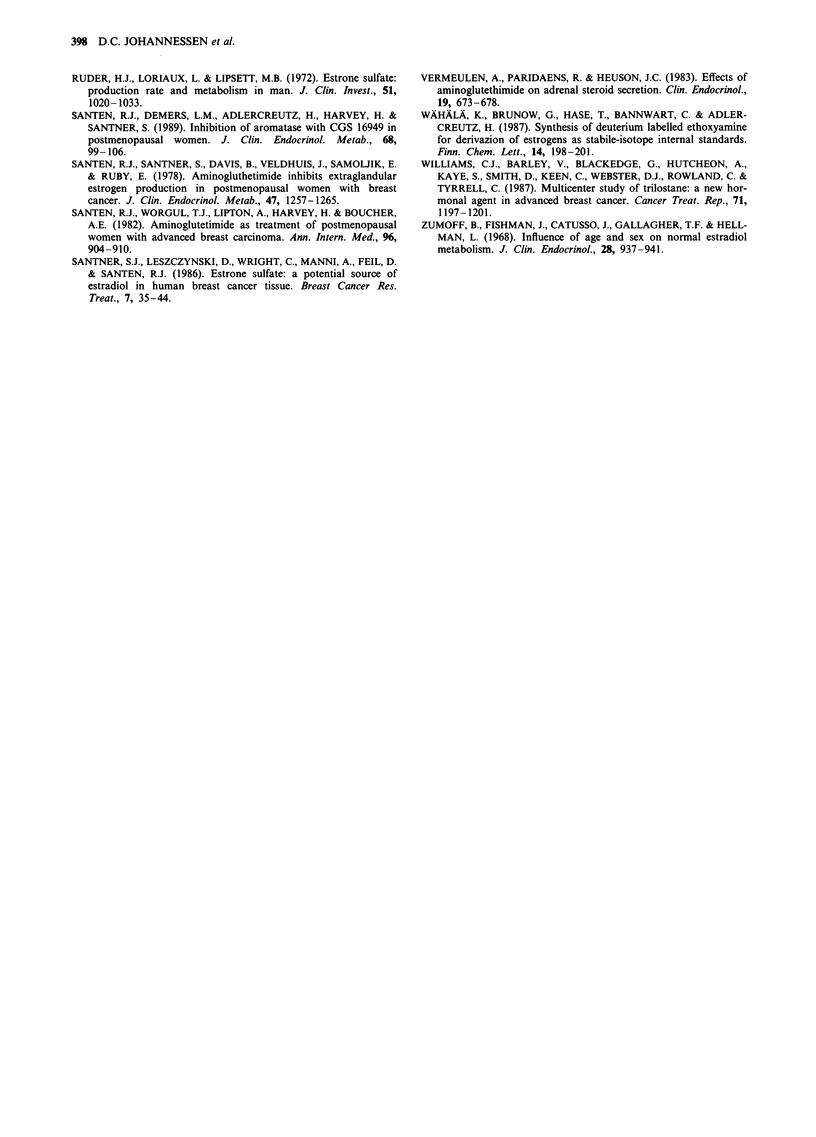# Plasma and urinary oestrogens in breast cancer patients on treatment with 4-hydroxyandrostenedione.

**DOI:** 10.1038/bjc.1993.347

**Published:** 1993-08

**Authors:** D. C. Johannessen, H. Adlercreutz, T. Fotsis, P. E. Lønning

**Affiliations:** Department of Oncology, University Hospital of Bergen, Haukeland sykehus, Norway.

## Abstract

Plasma and urinary oestrogens were measured in nine breast cancer patients (eight postmenopausal women and one man) before and during treatment with the aromatase inhibitor 4-hydroxyandrostenedione. Urinary oestrogens were measured by using a highly specific GC-MS method. Plasma levels of oestrone, oestradiol and oestrone sulphate were suppressed by 66.6% (+/- 3.6%), 57.7% (+/- 5.1%) and 51.8% (+/- 6.4%) respectively (P < 0.005 for all). Twenty-four hour urinary excretion of total oestrogens, oestradiol, oestriol, 2-hydroxyoestrone, 16 alpha-hydroxyoestrone and the minor metabolites 16 beta- and 15 alpha-hydroxyoestrone were all suppressed by mean values ranging from 60% to 82%, (oestradiol: P < 0.025, otherwise P < 0.005). There were no significant changes in the ratios between the different plasma oestrogens. The finding of sustained plasma and urinary oestrogens at 20-40% compared to their control levels indirectly support a hypothesis of alternative oestrogen sources in postmenopausal breast cancer patients on treatment with 4-hydroxyandrostenedione.


Br. J. Cancer (1993), 68, 393-398 © Macmillan Press Ltd., 1993

### Plasma and urinary oestrogens in breast cancer patients on treatment with 4-hydroxyandrostenedione

D.C. Johannessen[1], H. Adlercreutz[2], T. Fotsis[2]* & P.E. Lønning[1]

[1]Department of Oncology, University Hospital of Bergen, N-5021 Haukeland sykehus, Norway; and [2]Department of Clinical Chemistry, University of Helsinki, Meilathi Hospital, SF-00290 Helsinki, Finland.

**Summary** Plasma and urinary oestrogens were measured in nine breast cancer patients (eight postmenopausal women and one man) before and during treatment with the aromatase inhibitor 4-hydroxyandrostenedione. Urinary oestrogens were measured by using a highly specific GC-MS method. Plasma levels of oestrone, oestradiol and oestrone sulphate were suppressed by 66.6% (± 3.6%), 57.7% (± 5.1%) and 51.8% (± 6.4%) respectively ($P<0.005$ for all). Twenty-four hour urinary excretion of total oestrogens, oestradiol, oestriol, 2-hydroxyoestrone, 16α-hydroxyoestrone and the minor metabolites 16β - and 15α-hydroxyoestrone were all suppressed by mean values ranging from 60% to 82%, (oestradiol: $P<0.025$, otherwise $P<0.005$). There were no significant changes in the ratios between the different plasma oestrogens. The finding of sustained plasma and urinary oestrogens at 20-40% compared to their control levels indirectly support a hypothesis of alternative oestrogen sources in postmenopausal breast cancer patients on treatment with 4-hydroxyandrostenedione.

The aim of contemporary endocrine treatment of advanced breast cancer is to reduce oestrogen stimulation to the tumour cell. This could be achieved either by blocking oestrogen action at the receptor level with antioestrogens or by reducing the oestrogen supply to the tumour cell.

The major pathway of oestrogen production in postmenopausal women is peripheral conversion (aromatisation) of circulating androstenedione (A) into oestrone ($Oe_1$) (Grodin *et al.*, 1973). Aromatase inhibition is a successful approach to achieve plasma oestrogen suppression and tumour shrinkage in postmenopausal women suffering from breast cancer. The 'classic' aromatase inhibitor, aminoglutethimide (Orimetene®), has been in clinical use for more than two decades (Lønning & Kvinnsland, 1988). The toxic side effects caused by this drug has prompted the development of new and more selective aromatase inhibitors (Lønning *et al.*, 1990).

4-Hydroxyandrostenedione (Formestane®, Ciba-Geigy) is a second generation aromatase inhibitor (Brodie *et al.*, 1977), first reported to cause tumour shrinkage in breast cancer patients in 1984 (Coombes *et al.*, 1984). In contrast to aminoglutethimide, 4-hydroxyandrostenedione seems to act specifically on the aromatase enzyme (Brodie *et al.*, 1981; Dowsett *et al.*, 1989). The drug causes few side-effects, and results from phase I and II trials including more than 500 patients have revealed an overall response rate of 26% among unselected patients (Lønning, 1992).

The biochemical action of aromatase inhibitors *in vivo* is still incompletely understood. Different aromatase inhibitors like aminoglutethimide, 4-hydroxyandrostenedione (Formestane®, Ciba-Geigy) and CGS 16949 (Fadrazole®, Ciba-Geigy) inhibit aromatisation by 82-98% (Dowsett *et al.*, 1985; Jones *et al.*, 1992; Lønning *et al.*, 1991; Reed *et al.*, 1990; Santen *et al.*, 1978 ). Despite this, several investigators have reported sustained plasma oestrogens at about 30-50% of their control values in treated patients (Dowsett *et al.*, 1989; Dowsett *et al.*, 1990; Lønning *et al.*, 1989*b*; Santen *et al.*, 1982; Santen *et al.*, 1989; Vermeulen *et al.*, 1983). Some aromatase inhibitors may act on plasma oestrogen by mechanisms other than aromatase inhibition; aminoglutethimide has been shown to stimulate the metabolism of plasma oestrone sulphate ($Oe_1S$) by enhancing the production of 16α-hydroxylated metabolites (16α-hydroxyoestrone and oestriol) (Lønning *et al.*, 1987; Lønning *et al.*, 1989*a*; Lønning & Skulstad, 1989).

So far most studies have evaluated the influence of aromatase inhibitors on plasma oestrogens. However, due to the low levels of plasma oestrogens in patients on treatment with aromatase inhibitors, these steroids can be measured with RIA methods only, in which case there is a risk of non-specific interactions in the assay. About 60-70% of the oestrogen metabolites are excreted in the urine (Fishman *et al.*, 1966; Zumoff *et al.*, 1968), and the concentration of urinary oestrogen metabolites is about 100 times the concentration of plasma oestrogens. These urinary oestrogen metabolites may therefore be measured by highly specific GC-MS methods (Fotsis & Adlercreutz, 1987).

To our knowledge neither plasma $Oe_1S$ nor urinary oestrogen metabolites have previously been reported in patients treated with 4-hydroxyandrostenedione. This study was designed to test whether sustained plasma $Oe_1$ and $Oe_2$ levels in breast cancer patients treated with 4-hydroxyandrostenedione are accompanied by sustained plasma $Oe_1S$ and urinary oestrogen metabolite excretion. By comparing plasma and urinary oestrogen suppression, non specific interactions in the RIA assay or alteration in oestrogen disposition not related to aromatase inhibition might be reflected in conflicting results.

### Patients and methods

### *Patients*

Nine patients with advanced breast cancer (one man and eight postmenopausal women) who were to receive 4-hydroxyandrostenedione because of progressive disease were included in this study. All gave their verbal informed consent. The mean age was 72 years (range 62 to 79 years). No patients were smokers, and none of them received any other hormonal treatment or drugs known to influence drug metabolism. The liver enzymes and plasma creatinine were within the normal range in all patients, indicating normal liver and renal function.

All patients had previously been treated with two or more different endocrine regimens (range two to eight, median

Correspondence: E. Lønning, Department of Oncology, University Hospital of Bergen, N-5021 Haukeland Sykehus, Bergen, Norway.
*Present address: Department of Oncology and Immunology, Children's University Hospital, Ruprechts-Karls-Universität, INF 150, 6900 Heidelberg, Germany.
This work was partly presented at Sixth European Conference on Clinical Oncology and Cancer Nursing (ECCO 6), Firenze, October 1991, and European Society for Medical Oncology XVIIth Congress (ESMO XVII), Lyon, November 1992.
Received 16 December 1992; and in revised form 18 March 1993.

three regimens). Any previous hormonal therapy was terminated at least 4 weeks before commencing 4-hydroxyandrostenedione. Two patients had received previous treatment with aminoglutethimide, in which case aminoglutethimide was terminated 4 and 8 weeks before commencing on 4-hydroxyandrostenedione.

*Treatment schedule and sampling protocol*

All patients had i.m. injections of 4-hydroxyandrostenedione 250 mg. The injections were given weekly for the first 6 weeks, thereafter at 2-weekly intervals. Blood and urine sampling were performed before commencing on 4-hydroxyandrostenedione and after 36 to 80 days on treatment. The time interval from the last injection of 4-hydroxyandrostenedione to blood and urine collection in the on-treatment test situation ranged from 2 to 13 days with a median of 7 days.

Twenty-four hour urine was collected in dark glass bottles containing ascorbic acid (final concentration $>4\,g\,l^{-1}$) to prevent the catechol-oestrogens from undergoing oxidation (Gelbke, 1973). The urine was pooled, one aliquot was obtained for urinary creatinine measurement, and two aliquots of 50 ml were frozen and stored at −20°C until processing.

Heparinised blood samples were obtained on the morning of the urine collection period between 8 a.m. and 9 a.m. after an overnight fast. Plasma was separated by centrifugation and stored at −20°C until processing.

*Analytical methods*

Plasma oestrogens were measured by modification of RIA methods previously described (Dowsett *et al.*, 1987; Lønning *et al.*, 1989*a*). The sensitivity limit for $Oe_1$, $Oe_2$ and $Oe_1S$ was 2.1 pM, 6.3 pM and 25.9 pM respectively, and the Cv within assay were 4.4% 3.9% and 6%.

The analytical method for the determination of the urinary oestrogen profile based on capillary gas chromatography-mass spectrometry (GC/MS) in the selected ion monitoring (SIM) mode has been published (Fotsis & Adlercreutz, 1987). A significant improvement in the accuracy and precision of the method included the addition of deuterated ($d_5$)ethoxime derivatives (Wähälä *et al.*, 1987) of all ketonic oestrogens as internal standards immediately after hydrolysis of the urine extract. In this way stable-isotope dilution mass spectrometry could be used for all ketonic oestrogens. The final determination is carried out using a Hewlett Packard 5995B quadruple instrument equipped with a 0.2 mm × 12.4 m bonded phase BP 1 (equivalent to silicone SE-30) capillary silica column directly connected to ion source. The coefficients of variation for all fractions and other details regarding the reliability of the procedure have been published (Fotsis & Adlercreutz, 1987; Bannwart *et al.*, 1988). The following oestrogens were determined: Oestron e ($Oe_1$), Oestradiol ($Oe_2$), 2-hydroxy-oestrone (2-$OHOe_1$), 2-hydroxy-oestradiol (2-$OHOe_2$), 2-methoxyoestrone (2-$MeOOe_1$), 4-hydroxyoestrone (4-$OHOe_1$), oestriol ($Oe_3$), 16α-hydroxyoestrone (16α-$OHOe_1$), 16β-hydroxy-oestrone (16β-$OHOe_1$), 15α-hydroxyoestrone (15α-$OHOe_1$) and 16-keto-oestradiol (16-$KetoOe_2$).

Creatinine in urine and serum was measured by the method of Jaffé. As none of the patients had any significant change in plasma creatinine values during the investigation period, it was found feasible to use the creatinine clearance value as a 'recovery standard' for urine collection. Thus, to correct for any difference in urine losses between the two test situations, the amount of urinary oestrogens excreted was calculated using the ratio between the highest and the actual creatinine clearance for each patient as a correction factor.

*Statistical methods*

Plasma and urinary oestrogen levels before and during treatment were compared using the Wilcoxon Matched Pair Signed Rank Test. All *P*-values were expressed as two-tailed.

### Results

*Plasma oestrogens*

Plasma oestrogen levels before and during treatment are given in Table I and Figure 1. Treatment with 4-hydroxyandrostenedione suppressed plasma levels of $Oe_1$, $Oe_2$ and $Oe_1S$ in all patients ($P<0.005$). The mean percentage of suppression (± s.e.m.) was 66.6% (± 3.6%), 57.7% (± 5.1%) and 51.8% (± 6.4%) for $Oe_1$, $Oe_2$ and $Oe_1S$ respectively. While the $Oe_1/Oe_1S$ ratio decreased in eight of nine patients, this was not of statistical significance (a ratio of 0.213 ± 0.031 and 0.163 ± 0.027 before and during treatment respectively, $P = 0.080$). There was no change in the $Oe_2/Oe_1S$ ratio (0.042 ± 0.006 before and 0.037 ± 0.004 during treatment, $P>0.20$), but a small increase in the $O_2/Oe_1$ ratio (mean value before and during treatment 0.212 ± 0.022 and 0.260 ± 0.029 respectively; $P = 0.054$.)

*Urinary oestrogens*

Urinary excretion of total oestrogens and the different oestrogen metabolites is shown in Table II and in Figure 2a and 2b. The results may be summarised as follows:

(1) Urinary excretion of total oestrogens was suppressed by a mean value of 66%.
(2) All urinary metabolites except for 2-$OHOe_2$ and 4-$OHOe_1$ were significantly suppressed (mean values of suppression ranging from 60% to 82%). The urinary concentration of 4-$OHOe_1$ was below the sensitivity limit of the assay in the control situation. Thus, the result obtained for this metabolite should be interpreted with caution.
(3) No significant alterations in the ratio of the 16α-hydroxylated metabolites (16α-$OHOe_1$ and $Oe_3$) or 2-$OHOe_1$ relative to $Oe_1$ were found.

*Comparison of plasma and urinary oestrogen suppression*

The relative suppressions of urinary and plasma oestrogens were of the same magnitude. A suppression of total urinary oestrogen by 66% (± 5.6%) corresponds well to a suppression of plasma $Oe_1$, $Oe_2$ and $Oe_1S$ of 66.6 (± 3.6%), 57.7% (± 5.1%) and 51.8% (± 6.4%) respectively.

**Table I** Mean values in pmol $l^{-1}$ (± s.e.m.) and mean percentual suppression (± s.e.m.) before and during treatment with 4-hydroxyandrostenedione

| | *Before* | *During* | *% suppression* | *P* |
|---|---|---|---|---|
| Plasma Oe2 | 15.4 ± 3.4 | 5.5 ± 0.7 | 57.8 ± 5.1 | <0.005 |
| Plasma Oe1 | 70.6 ± 10.8 | 21.0 ± 1.5 | 66.6 ± 3.6 | <0.005 |
| Plasma Oe1S | 456.0 ± 131.1 | 197.0 ± 83.5 | 51.8 ± 6.4 | <0.005 |
| Ratio Oe2/Oe1S | 0.042 ± 0.006 | 0.037 ± 0.004 | | ns |
| Ratio Oe2/Oe1 | 0.212 ± 0.022 | 0.260 ± 0.029 | | 0.054 |
| Ratio Oe1/Oe1S | 0.213 ± 0.031 | 0.163 ± 0.027 | | 0.080 |

**Figure 1** Individual plasma levels of oestradiol P-$Oe_2$), oestrone (P-$Oe_1$) and oestrone sulphate (P-$Oe_1S$) before and during treatment in 9 breast cancer patients (8 postmenopausal women: O, and 1 man: ■) treated with 250 mg 4-hydroxyandrostenedione i.m. fortnightly.

### Discussion

Plasma levels of $Oe_1$, $Oe_2$ and $Oe_1S$ obtained in our patients before treatment were in the same range as previously reported by us and others for postmenopausal breast cancer patients but plasma $Oe_1S$ was in the low normal range (Dowsett *et al.*, 1989; Lønning *et al.*, 1989*b*; Vermeulen *et al.*, 1983). The amount of different urinary oestrogen metabolites excreted during 24 h was in the same range as previously reported in breast cancer patients (Aldercreutz *et al.*, 1991).

The relative suppression of plasma $Oe_1$ and $Oe_2$ obtained by 4-hydroxyandrostenedione was of the same magnitude as previously reported by others (Dowsett *et al.*, 1989; Reed *et al.*, 1990). To our knowledge, plasma $Oe_1S$ and urinary oestrogen metabolite excretion have not been measured in patients on treatment with 4-hydroxyandrostenedione previously. Oestrone sulphate has been suggested to play an important role as an oestrogen source to the tumour cell (Santner *et al.*, 1986; Pasqualini *et al.*, 1989), and the influence of aromatase inhibitors on plasma $Oe_1S$ levels may be of significant biological importance.

Aromatase is a key enzyme in postmenopausal oestrogen synthesis. Current opinion is that peripheral aromatisation of circulating A and testosterone (T) into $Oe_1$ and $Oe_2$ respectively accounts for total postmenopausal oestrogen synthesis.

**Figure 2a,b** Twenty-four hours urinary excretion of the major oestrogen metabolites and total urinary oestrogens before and during treatment in 9 breast cancer patients (8 postmenopausal women: O, and 1 man: ■) treated with 250 mg 4-hydroxyandrostenedione i.m. fortnightly. U-$Oe_2$ = urinary oestradiol, U-$Oe_1$ = urinary oestrone, U-$Oe_3$ = urinary oestriole. Total U-Oe = total urinary oestrogen, 2-$OHOe_1$ = 2-hydroxyoestrone and 16α-$OHOe_1$ = 16α-hydroxyoestrone.

**Table II** Mean values in nmol 24 $h^{-1}$ (± s.e.m.) and mean percentual suppression (± s.e.m.) of 24 h urinary metabolites before and during treatment with 4-hydroxyandrostenedione

| *Metabolite* | *Before* | *During* | *% suppression* | *P* |
|---|---|---|---|---|
| 2-OHOe1 | 4.64 ± 1.47 | 1.09 ± 0.25 | 70.8 ± 5.2 | <0.005 |
| 4-OHOe1 | 0.54 ± 0.15 | 0.43 ± 0.12 | 13.5 ± 16.0 | >0.2 |
| 2-OHOe2 | 2.27 ± 0.39 | 1.44 ± 0.37 | 14.2 ± 39.2 | >0.1 |
| Oe2 | 1.57 ± 0.27 | 0.44 ± 0.11 | 59.7 ± 17.4 | <0.025 |
| Oe1 | 5.86 ± 1.26 | 1.90 ± 0.35 | 63.6 ± 5.3 | <0.005 |
| 2-methoxyOe1 | 1.13 ± 0.22 | 0.28 ± 0.07 | 72.9 ± 4.2 | <0.005 |
| 16-OHOe1 | 1.91 ± 0.26 | 0.42 ± 0.07 | 78.1 ± 2.3 | <0.005 |
| 15-OHOe1 | 0.35 ± 0.12 | 0.10 ± 0.02 | 60.2 ± 8.2 | <0.005 |
| 16-OHOe1 | 1.30 ± 0.33 | 0.23 ± 0.12 | 81.8 ± 7.9 | <0.005 |
| 16-ketoOe2 | 1.04 ± 0.17 | 0.23 ± 0.09 | 74.3 ± 9.5 | <0.005 |
| Oe3 | 7.62 ± 1.90 | 1.92 ± 0.41 | 68.3 ± 8.3 | <0.005 |
| Total oestrogens | 28.24 ± 4.48 | 8.47 ± 1.37 | 66.2 ± 5.6 | <0.005 |

Aromatase inhibition is a successful treatment approach in postmenopausal breast cancer. Treatment with different aromatase inhibitors like aminoglutethimide, 4-hydroxyandrostenedione and CGS 16949A all cause effective suppression of plasma oestrogens (Santen *et al.*, 1982; Santen *et al.*, 1989; Dowsett *et al.*, 1989; Dowsett *et al.*, 1990) and clinical responses comparable to what may be expected from the most effective forms of endocrine treatment like antioestrogens and high dose progestins (Lønning *et al.*, 1992). Thus, aromatase inhibitors differ significantly from other drugs investigated as hormone suppressors in postmenopausal breast cancer. Glucocorticoids, ketoconazole and trilostane all suppress adrenal steroid synthesis, cause a modest suppression of plasma oestrogens, and produce clinical responses in a small number of patients (Harris *et al.*, 1988; Harris *et al.*, 1984; Beardwell *et al.*, 1983; Coombes *et al.*, 1985; Williams *et al.*, 1987). The results obtained with these drugs compared with aromatase inhibitors indirectly suggest a dose response relationship between plasma oestrogen suppression and the chance of achieving a clinical response in postmenopausal breast cancer patients. Accordingly, a major goal is to achieve maximal oestrogen suppression.

The efficacy of an aromatase inhibitor may be assessed in different ways. One approach is to measure *in vivo* aromatase inhibition by use of isotope tracer infusions (Jacobs *et al.*, 1991), another approach is to measure the degree of plasma oestrogen suppression. A major problem is to explain the inconsistency of the results obtained with these different methods and to interpret the finding of sustained plasma oestrogens despite subtotal aromatase inhibition. There are two possible explanations for these findings. First, they may be due to technical flaws in the tracer infusion studies or with plasma oestrogen analysis. Second, they indicate some alternative sources of plasma oestrogens in patients on treatment with aromatase inhibitors.

Considering the first possibility, tracer studies have revealed a $>90\%$ inhibition of the conversion of circulating androstenedione into oestrone during treatment with 4-hydroxyandrostenedione (Jones *et al.*, 1992; Reed *et al.*, 1990) as well as with other aromatase inhibitors (Lønning *et al.*, 1991; McNeill *et al.*, 1992; Santen *et al.*, 1978). These methods are sensitive enough to detect aromatase inhibition down to 98–99% (Jacobs *et al.*, 1991). Any non-specific interaction in the chromatograms may be expected to cause an underestimation of the degree of inhibition. Thus, it is not likely that these studies may have overrated the efficacy of these drugs. While the possibility of non-specific interactions in the radioimmunoassays is a more likely event, our finding of an internal consistency between the relative suppression of plasma oestrogens measured by RIA techniques and the suppression of urinary oestrogen metabolites measured by a specific GC-MS method provides indirect evidence this may not be the case.

The possibility of alternative oestrogen sources in breast cancer patients on treatment with aromatase inhibitors should be considered. These could be enzymatic pathways not inhibited by current drugs or, alternatively, that the oestrogen synthesis could partly take place in compartments not equilibrating with circulating androstenedione or not penetrated by aromatase inhibitors. So far there is no direct evidence pointing to any such a pathway. Alternatively, plasma oestrogens could be derived from residual tissue oestrogens, like $Oe_1S$ or lipoidal oestrogen conjugates (Larner *et al.*, 1992) which may have a slow turnover and could be sustained in the tissue for a long time even when their production is inhibited. While this possibility can not be excluded, we found no correlation between plasma oestrogen suppression and the duration of 4-hydroxyandrostenedione treatment among patients investigated in this study. Results by others (Dowsett *et al.*, 1985*b*) as well as unpublished data from our group suggest plasma oestrogens to be sustained also in patients who have been on aminoglutethimide treatment for more than 6 months. While it is not possible at this stage to draw any conclusion considering possible sources of these oestrogens, our finding of an internal consistency between the relative suppression of plasma and urinary oestrogens in breast cancer patients treated with 4-hydroxyandrostenedione add indirect support to a hypothesis that the sustained plasma oestrogens are real oestrogens and not technical artefacts. This finding may have significant implications for future studies on aromatase inhibitors in the treatment of breast cancer.

The major metabolic pathways of oestrogens are hydroxylation in the 2- and 16α-position (Bolt, 1979). Our results revealed no significant change in the ratio of the 16α-hydroxylated (16α-$OHOe_1$ and $Oe_3$) or 2-hydroxylated (2-$OHOe_1$) metabolites to $Oe_1$ in the urine. Thus, in contrast to aminoglutethimide (Lønning *et al.*, 1989*a*; Lønning & Skulstad, 1989) 4-hydroxyandrostenedione does not seem to influence the major oestrogen metabolic pathways *in vivo*.

The metabolite 4-$OHOe_1$, like 2-$OHOe_2$, was little suppressed by treatment with 4-hydroxyandrostenedione. The possibility exists that urinary 4-$OHOe_1$ could arise from aromatisation of 4-hydroxyandrostenedione. However, as the urinary level of 4-$OHOe_1$ before treatment was below the detection limit of the method, the result should be interpreted with caution. The contribution of 4-$OHOe_1$ to total urinary oestrogens before and during 4-hydroxyandrostenedione treatment was 1.6% and 3.8% only. Thus, under any circumstance a direct production of 4-$OHOe_1$ from 4-hydroxyandrostenedione most probably would not be of a magnitude of biological importance and may not explain our finding of sustained plasma and urinary oestrogens.

The reason why 2-$OHOe_2$ is relatively moderately suppressed remains unclear. At these low levels, the assay may not be completely specific. The urinary concentration of this metabolite was low. While there was a small increase in the plasma $Oe_2/Oe_1$ ratio, the ratio of $Oe_2/Oe_1$ in urine was slightly reduced. While previous investigations have suggested a possible influence by 4-hydroxyandrostenedione on the 17β-hydroxysteroid dehydrogenase *in vitro* (Brodie *et al.*, 1982), our results do not suggest any influence of 4-hydroxyandrostenedione on this enzyme *in vivo*. Neither is there any obvious reason why 16β-$OHOe_1$ was particularly effective suppressed (mean suppression of 82%). However, it should be considered that oestrogen metabolic enzymes have been shown to be influenced by several exogenous factors (Conney, 1986), and our current knowledge of the regulation of these enzymes is incomplete.

Considering the plasma oestrogens, it may be noted that the plasma $Oe_1/Oe_1S$ ratio was reduced in 8/9 patients. This contrasts the findings obtained with aminoglutethimide in which case the $Oe_1/Oe_1S$ ratio was elevated due to enhancement of $Oe_1S$ metabolism (Lønning *et al.*, 1989*b*). Our findings seem to exclude any enhancement of $Oe_1S$ metabolism by 4-hydroxyandrostenedione, but further studies are needed to assess whether treatment with 4-hydroxyandrostenedione causes a reduction in the $Oe_1/Oe_1S$ ratio. If this should be the case, it may provide information of clinical importance. Current opinion in the literature is that there is no evidence of a direct secretion of plasma $Oe_1S$ in postmenopausal women, as circulating $Oe_1S$ seems to be accounted for by production from plasma $Oe_1$ and $Oe_2$ (Longcope *et al.*, 1972; Ruder *et al.*, 1972; Lønning *et al.*, 1989*a*). However, it should be recalled that patients on treatment with aromatase inhibitors have plasma oestrogens markedly lower than other postmenopausal women. Accordingly, while a small secretion of $Oe_1S$ could be difficult to detect in postmenopausal women in general, it could be of importance in patients having their major oestrogen production pathways blocked by an aromatase inhibitor. More studies are needed to evaluate this phenomenon, but our findings underline the importance of measuring plasma $Oe_1S$ in concert with $Oe_1$ and $Oe_2$ in patients treated with aromatase inhibitors.

### Conclusions

4-Hydroxyandrostenedione suppresses plasma and urinary oestrogens in breast cancer patients, but the finding that both plasma and urinary oestrogens remained at levels 30-50% of their control values supports the hypothesis that alternative oestrogen sources may exist in breast cancer patients. In contrast to aminoglutethimide, 4-hydroxyandrostenedione does not seem to have any major influence on the major pathways of oestrogen metabolism.

This work was supported by grants from the Norwegian Cancer Society, the Sigrid Jusélius Foundation, the Finnish Cancer Foundation and Nordisk Cancer Union. The technical assistance of Mr D. Ekse and Mrs Anja Koskela, Mrs Juga Wiik and Mrs Sirkka Adlercreutz is greatly appreciated. Dr Johannessen is a research fellow of the Norwegian Cancer Society. The study drug was a gift from Ciba Geigy.

### References

ADLERCREUTZ, H., FOTSIS, T., HÖCKERSTEDT, K., PELONEN, U. & OLLUS, A. (1991). Breast cancer and estrogens: a study in old women. *Fourth International Congress on Hormones and Cancer*. Amsterdam, The Nederlands Sept. 15-19.

BANNWART, C., ADLERCREUTZ, H., WÄHÄLÄ, K., BRUNOW, G. & HASE, T. (1987). Deuterium labelled ethoximes as stable isotope internal standards in the GC/MS-SIM determination of oxo-estrogens in human urine extracts: preliminary results. In *Advances in Steroid Analyses '87*, Görög, S. & Heftmann, E., (ed.). Proceedings of the Symposium on the analyses of Steroids, Sopron, Hungary, October 20-22, 1987. Budapest Akadémiai Kiadó, 1988: 283-286.

BEARDWELL, C.G., HINDLEY, A.C., WILKINSON, P.M., TODD, I.D.H., RIBEIRO, C.G. & BU'LOCK, D. (1983). Trilostane in the treatment of advanced breast cancer. *Cancer Chemoter. Pharmacol.*, **10**, 158-162.

BOLT, H.M. (1979). Metabolism of estrogens - natural and synthetic. *Pharmac. Ther.*, **4**, 155-181.

BRODIE, A.M.H., GARRETT, W.M., HENDRICKSON, J.R., TSAI-MORRIS, C.H., MARCOTTE, P.A. & ROBINSON, C.H. (1981). Inactivation of aromatase *in vitro* by 4-hydroxyandrostenedione and 4-acetooxyandrostenedione and sustained effects *in vivo*. *Steroids*, **38**, 693-702.

BRODIE, A.M.H., BRODIE, H.J., ROMANOFF, L., WILLIAMS, J.G., WILLIAMS, K.I.H. & WU, J.T. (1982). Inhibition of biosynthesis and regression of mammary tumours by aromatase inhibitors. *Hormones Cancer Adv. Exp. Med. Biol.*, **138**, 179-190.

BRODIE, A.M.H., SCHWARZEL, W.C., SAIKH, A.A. & BRODIE, H.J. (1977). The effect of an aromatase inhibitor, 4-hydroxy-4-androstene-3,17-dione, on estrogen dependent processes in reproduction and breast cancer. *Endocrinology*, **100**, 1684-1695.

CONNEY, A.H., (1986). Induction of microsomal cytochrome P-450 enzymes: the first Bernhard B. Brodie lecture at Pennsylvania State University. *Life Sciences*, **39**, 2493-2518.

COOMBES, R.C., GOSS, P., DOWSETT, M., GAZET, J.C. & BRODIE, A.M.H. (1984). 4-Hydroxyandrostenedione in treatment of post-menopausal patients with advanced breast cancer. *Lancet*, **ii**, 1237-1239.

COOMBES, R.C., POWLES, T.J., MUINDI, J., HUNT, J., WARD, M., PEREZ, D. & NEVILLE, A.M. (1985). Trilsotane therapy for advanced breast cancer. *Cancer Treat. Rep.*, **69**, 351-354.

DOWSETT, M., CUNNINGHAM, D.C., STEIN, R.C., EVANS, S., DEHENNIN, L., HEDLEY, A. & COOMBES, R.C. (1989). Dose related endocrine effects and pharmacokinetics of oral and intra-muscular 4-hydroxyandrostenedione in postmenopausal breast cancer patients. *Cancer Res.*, **49**, 1306-1312.

DOWSETT, M., GOSS, P.E., POWLES, T.J., HUTCHINSON, G., BRODIE, A.M., JEFFCOATE, S.L. & COOMBES, R.C. (1987). Use of the aromatase inhibitor 4-hydroxyandrostenedione in postmeno-pausal breast cancer: optimization of therapeutic dose and route. *Cancer Res.*, **47**, 1957-1961.

DOWSETT, M., HARRIS, A.L., STUART-HARRIS, R., HILL, M., CANT-WELL, B.M.J., SMITH, I.E. & JEFFCOATE, S.L. (1985). A comparison of the endocrine effects of low dose aminoglutethimide with and without hydrocortisone in postmenopausal breast cancer patients. *Br. J. Cancer*, **52**, 525-529.

DOWSETT, M., SANTNER, S.J., SANTEN, R.J., JEFFCOATE, S.L. & SMITH, I.E. (1985). Effective inhibition of low dose amino-glutethimide of peripheral aromatization in postmenopausal breast cancer patients. *Br. J. Cancer*, **52**, 31-35.

DOWSETT, M., STEIN, R.C., METHA, A. & COOMBES, R.C. (1990). Potency and selectivity of the non-steroidal aromatase inhibitor CGS 16949A in post menopausal breast cancer patients. *Clin. Endocrinol.*, **32**, 623-634.

FISHMAN, J., HELLMAN, L., ZUMOFF, B. & CATUSSO, J. (1966). Pathway and stereochemistry of the formation of estiols in man. *Biochemistry*, **5**, 1794-1798.

FOTSIS, T. & ADLERCREUTZ, H. (1987). The multicomponent analysis of estrogens in urine by ion exchange chromatography GC-MS-I. Quantitation of oestrogens after initial hydrolysis of conjugates. *J. Steroid Biochem.*, **28**, 203-213.

GRODIN, J.M., SIITERI, P.K. & MACDONALD, P.C. (1973). Source of estrogen production in postmenopausal women., *J. Clin. Endocrinol. Metab.*, **36**, 207-214.

HARRIS, A.L., CANTWELL, B.M.J. & DOWSETT, M. (1988). High dose ketoconazole: endocrine and therapeutic effects in postmeno-pausal breast cancer. *Br. J. Cancer*, **58**, 493-496.

HARRIS, A.L., DOWSETT, M., SMITH, I.E. & JEFFCOATE, S.L. (1984). Hydrocortisone *vs* hydrocortisone plus aminoglutethimide: a comparison of the endocrine effects in postmenopausal breast cancer. *Eur. J. Cancer Clin. Oncol.*, **20**, 463-469.

JACOBS, S., LØNNING, P.E., HAYNES, B., GRIGGS, L. & DOWSETT, M. (1991). Measurement of aromatisation by a urine technique suitable for the evaluation of aromatase inhibitors *in vivo*. *J. Enzyme Inhib.*, **4**, 315-325.

JONES, A.L., MACNEILL, F., JACOBS, S., LØNNING, P.E., DOWSETT, M. & POWLES, T.J. (1992). The influence of intramuscular 4-hydroxyandrostenedione on peripheral aromatisation in breast cancer patients. *Eur. J. Cancer*, **28A**, 1712-1716.

LARNER, J.M., SCHACKLETON, C.H.L., ROITMAN, E., SCHWARTZ, P.E. & HOCHBERG, R.B. (1992). Measurement of estradiol-17-fatty acid esters in human tissue. *J. Clin. Endocrinol. Metabol.*, **75**, 195-200.

LONGCOPE, C. (1972). The metabolism of estrone sulphate in normal males. *J. Clin. Endocrinol. Metabol.*, **34**, 113-122.

LØNNING, P.E. (1992). Aromatase inhibition: past, present and future. In *Endocrine Aspects of Breast Cancer*, Dowsett, M. (ed.), pp. 53-75. The Parthenon Publishing Group Ltd: Carnforth, UK.

LØNNING, P.E., DOWSETT, M. & POWLES, T.J. (1990). Postmeno-pausal estrogen synthesis and metabolism: alteration caused by aromatase inhibitors used for the treatment of breast cancer. *J. Steroid Biochem.*, **35**, 335-366.

LØNNING, P.E., JACOBS, S., JONES, A., HAYNES, B., POWLES, T.J. & DOWSETT, M. (1991). The influence of CGS 16949A on peripheral aromatisation in breast cancer patients. *Br. J. Cancer*, **63**, 789-793.

LØNNING, P.E., JOHANNESSEN, D.C. & THORSEN, T. (1989*a*). Alterations in the production rate and the metabolism of oestrone and oestrone sulfate in breast cancer patients treated with amino-glutethimide. *Br. J. Cancer*, **60**, 107-111.

LØNNING, P.E., JOHANNESSEN, D.C., THORSEN, T. & EKSE, D. (1989*b*). Effects of aminoglutethimide on plasma estrone sulfate not caused by aromatase inhibition. *J. Steroid Biochem.*, **33**, 541-545.

LØNNING, P.E. & KVINNSLAND, S. (1988). Mechanisms of action of aminoglutethimide as endocrine therapy of breast cancer. *Drugs*, **35**, 685-710.

LØNNING, P.E., KVINNSLAND, S., THORSEN, T. & UELAND, P.M. (1987). Alterations in the metabolism of oestrogens during treatment with aminoglutethimide in breast cancer patients. Preliminary findings. *Clin. Pharmakokinet.*, **13**, 393-406.

LØNNING, P.E., LIEN, E., LUNDGREN, S. & KVINNSLAND, S. (1992*a*). Disposition of new endocrine drugs used in the treatment of advanced breast cancer. *Clin. Pharmacokinet.*, **22**, 327-358.

LØNNING, P.E. & SKULSTAD, P. (1989). Alteration in the urine excretion of estrogen metabolites in breast cancer women treated with aminoglutethimide. *J. Steroid Biochem.*, **33**, 565-571.

MACNEILL, F.A., JONES, A.L., JACOBS, S., LØNNING, P.E., POWLES, T.J. & DOWSETT, M. (1992). The influence of Aminoglutethimide and its analogue Rogletimide on peripheral aromatisation in breast cancer. *Br. J. Cancer*, **66**, 692-697.

PASQUALINI, J.R., GELLY, C., NGUYEN, B.-L. & VELLA, C. (1989). Importance of estrogen sulfates in breast cancer. *J. Steroid Biochem.*, **34**, 155-163.

REED, M.J., LAI, L.C., OWEN, A.M., SINGH, A., COLDHAM, N.G., PUHROHIT, A., GHILCHIK, M.W., SHAIKH, N.A. & JAMES, V.H.T. (1990). Effect of treatment with 4-hydroxyandrosetenedione on the peripheral conversion of androsestendione to estrone and *in vitro* tumor aromatase activity in postmenopausal breast cancer. *Cancer Res.*, **50**, 193-196.

RUDER, H.J., LORIAUX, L. & LIPSETT, M.B. (1972). Estrone sulfate: production rate and metabolism in man. *J. Clin. Invest.*, **51**, 1020–1033.

SANTEN, R.J., DEMERS, L.M., ADLERCREUTZ, H., HARVEY, H. & SANTNER, S. (1989). Inhibition of aromatase with CGS 16949 in postmenopausal women. *J. Clin. Endocrinol. Metab.*, **68**, 99–106.

SANTEN, R.J., SANTNER, S., DAVIS, B., VELDHUIS, J., SAMOLJIK, E. & RUBY, E. (1978). Aminogluthetimide inhibits extraglandular estrogen production in postmenopausal women with breast cancer. *J. Clin. Endocrinol. Metab.*, **47**, 1257–1265.

SANTEN, R.J., WORGUL, T.J., LIPTON, A., HARVEY, H. & BOUCHER, A.E. (1982). Aminoglutetimide as treatment of postmenopausal women with advanced breast carcinoma. *Ann. Intern. Med.*, **96**, 904–910.

SANTNER, S.J., LESZCZYNSKI, D., WRIGHT, C., MANNI, A., FEIL, D. & SANTEN, R.J. (1986). Estrone sulfate: a potential source of estradiol in human breast cancer tissue. *Breast Cancer Res. Treat.*, **7**, 35–44.

VERMEULEN, A., PARIDAENS, R. & HEUSON, J.C. (1983). Effects of aminoglutethimide on adrenal steroid secretion. *Clin. Endocrinol.*, **19**, 673–678.

WÄHÄLÄ, K., BRUNOW, G., HASE, T., BANNWART, C. & ADLERCREUTZ, H. (1987). Synthesis of deuterium labelled ethoxyamine for derivazion of estrogens as stabile-isotope internal standards. *Finn. Chem. Lett.*, **14**, 198–201.

WILLIAMS, C.J., BARLEY, V., BLACKEDGE, G., HUTCHEON, A., KAYE, S., SMITH, D., KEEN, C., WEBSTER, D.J., ROWLAND, C. & TYRRELL, C. (1987). Multicenter study of trilostane: a new hormonal agent in advanced breast cancer. *Cancer Treat. Rep.*, **71**, 1197–1201.

ZUMOFF, B., FISHMAN, J., CATUSSO, J., GALLAGHER, T.F. & HELLMAN, L. (1968). Influence of age and sex on normal estradiol metabolism. *J. Clin. Endocrinol.*, **28**, 937–941.