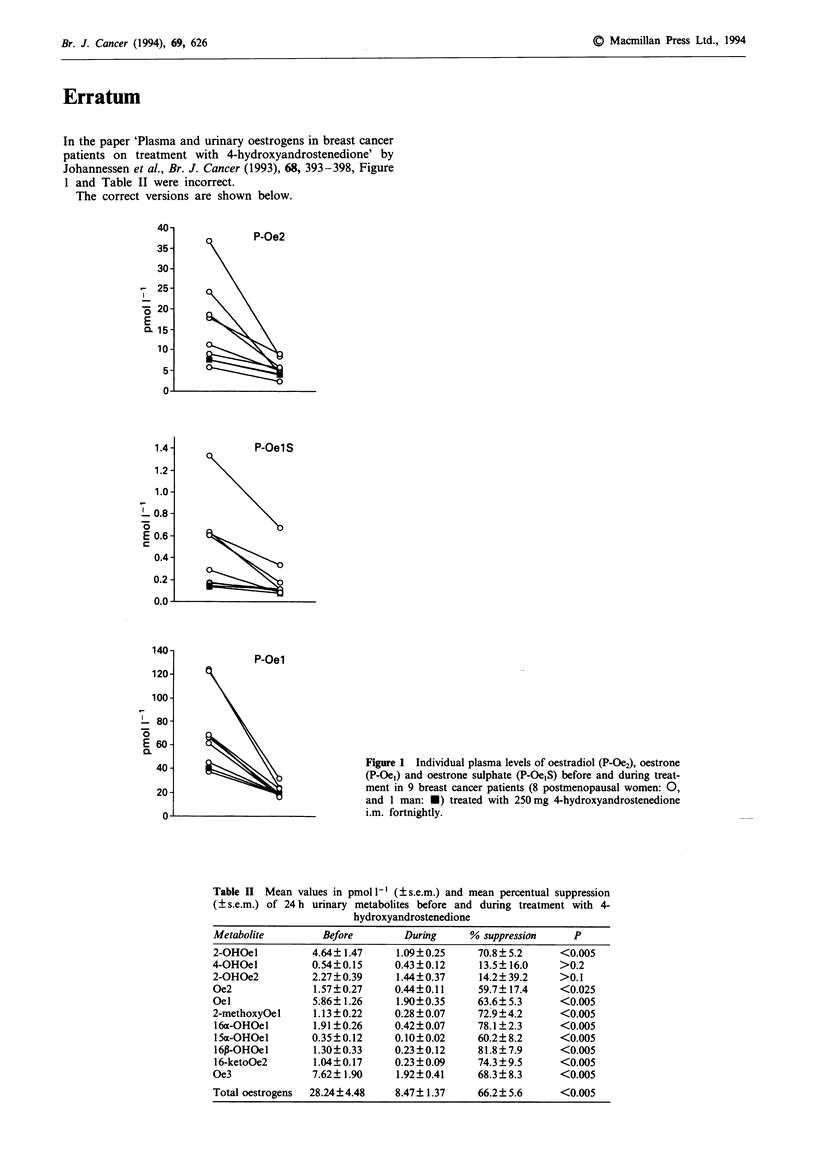# Erratum

**Published:** 1994-03

**Authors:** 


 © Macmillan Press Ltd., 1994

### Erratum

In the paper 'Plasma and urinary oestrogens in breast cancer patients on treatment with 4-hydroxyandrostenedione' by Johannessen *et al.*, *Br. J. Cancer* (1993), **68**, 393–398, Figure 1 and Table II were incorrect.

The correct versions are shown below.

**Figure 1** Individual plasma levels of oestradiol (P-$Oe_2$), oestrone (P-$Oe_1$) and oestrone sulphate (P-$Oe_1$S) before and during treatment in 9 breast cancer patients (8 postmenopausal women: O, and 1 man: ■) treated with 250 mg 4-hydroxyandrostenedione i.m. fortnightly.

**Table II** Mean values in pmol $l^{-1}$ (±s.e.m.) and mean percentual suppression (±s.e.m.) of 24 h urinary metabolites before and during treatment with 4-hydroxyandrostenedione

| *Metabolite* | *Before* | *During* | *% suppression* | *P* |
|---|---|---|---|---|
| 2-OHOe1 | 4.64±1.47 | 1.09±0.25 | 70.8±5.2 | <0.005 |
| 4-OHOe1 | 0.54±0.15 | 0.43±0.12 | 13.5±16.0 | >0.2 |
| 2-OHOe2 | 2.27±0.39 | 1.44±0.37 | 14.2±39.2 | >0.1 |
| Oe2 | 1.57±0.27 | 0.44±0.11 | 59.7±17.4 | <0.025 |
| Oe1 | 5:86±1.26 | 1.90±0.35 | 63.6±5.3 | <0.005 |
| 2-methoxyOe1 | 1.13±0.22 | 0.28±0.07 | 72.9±4.2 | <0.005 |
| 16α-OHOe1 | 1.91±0.26 | 0.42±0.07 | 78.1±2.3 | <0.005 |
| 15α-OHOe1 | 0.35±0.12 | 0.10±0.02 | 60.2±8.2 | <0.005 |
| 16β-OHOe1 | 1.30±0.33 | 0.23±0.12 | 81.8±7.9 | <0.005 |
| 16-ketoOe2 | 1.04±0.17 | 0.23±0.09 | 74.3±9.5 | <0.005 |
| Oe3 | 7.62±1.90 | 1.92±0.41 | 68.3±8.3 | <0.005 |
| Total oestrogens | 28.24±4.48 | 8.47±1.37 | 66.2±5.6 | <0.005 |